# Role of HNF4alpha-cMyc interaction in liver regeneration after partial hepatectomy

**DOI:** 10.3389/fendo.2024.1404318

**Published:** 2024-07-31

**Authors:** Manasi Kotulkar, Diego Paine-Cabrera, Kaitlyn Venneman, Udayan Apte

**Affiliations:** Department of Pharmacology, Toxicology, and Therapeutics, University of Kansas Medical Center, Kansas City, KS, United States

**Keywords:** HNF4α, proliferation, partial hepatectomy, regeneration, progenitor cells

## Abstract

**Background:**

Hepatocyte nuclear factor 4 alpha (HNF4α) is the master regulator of hepatic differentiation. Recent studies have also revealed the role of HNF4α in hepatocyte proliferation via negatively regulating the expression of proto-mitogenic genes, including cMyc. Here, we aimed to study the interaction between HNF4α-cMyc during liver regeneration after partial hepatectomy (PHX).

**Methods:**

Wild-type (WT), hepatocyte-specific knockout of HNF4α (HNF4α-KO), cMyc (cMyc-KO), and HNF4α-cMyc double knockout (DKO) mice were subjected to PHX to induce liver regeneration. Blood and liver tissue samples were collected at 0h, 24h, 48h, 7D, and 14D after PHX for further analysis.

**Results:**

WT, HNF4α-KO, cMyc-KO and DKO mice regained liver weight by 14 days after PHX. The deletion of cMyc did not affect liver regeneration, which was similar to the WT mice. WT and cMyc-KO mice started regaining liver weight as early as 24 hours after PHX, with a peak proliferation response at 48 hours after PHX. HNF4α- KO and DKO showed a delayed response with liver weight increase by day 7 after PHX. The overall hepatocyte proliferation response by DKO mice following PHX was lower than that of other genotypes. Interestingly, the surviving HNF4α-KO and DKO mice showed re-expression of HNF4α at mRNA and protein levels on day 14 after PHX. This was accompanied by a significant increase in the expression of *Krt19* and *Epcam*, hepatic progenitor cell markers, in the DKO mice on day 14 after PHX.

**Conclusion:**

These data indicate that, in the absence of HNF4α, cMyc contributes to hepatocyte-driven proliferation to compensate for the lost tissue mass. Furthermore, in the absence of both HNF4α and cMyc, HPC-driven proliferation occurs to support liver regeneration.

## Introduction

Hepatocyte nuclear factor 4 alpha (HNF4α) is a highly conserved member of the nuclear receptor expressed at high levels in hepatocytes. HNF4α is important for normal liver development and the maintenance of hepatic differentiation (1). HNF4α regulates various metabolic processes, including bile acid and coagulation factor synthesis, lipid, glucose and amino acid metabolism, and expression of several drug metabolism genes (2–5). Recent studies have shown that HNF4α also regulates hepatocyte proliferation (6). Deletion of HNF4α results in increased spontaneous hepatocyte proliferation without liver injury, and it also promotes carcinogen-induced hepatocellular carcinoma (6). HNF4α negatively regulates several pro-mitogenic genes, including but not limited to the proto-oncogene cMyc (7).

The liver has an exceptional ability to regenerate following drug-and diet-induced liver injury and surgical resection (8). Liver regeneration is a highly regulated process that involves cell proliferation and tissue remodeling. Partial hepatectomy (PHX) is the most common model for studying liver regeneration, in which approximately 70% of the liver is surgically removed and the remnant liver is allowed to regenerate (9). After PHX, hepatocytes leave the quiescence phase and start proliferating to contribute to the regeneration process. This model is clinically significant because PHX is a common therapy for several chronic liver diseases and is also the basis for successful living donor liver transplantation (10). Furthermore, understanding the mechanisms of the initiation and termination of hepatocyte proliferation is crucial because excessive proliferation can lead to carcinogenesis.

Previous studies in our laboratory investigated the role of HNF4α in regulating liver regeneration after PHX (7). Our studies revealed that HNF4α is essential for the termination of liver regeneration. Other studies from our laboratory investigated the role of HNF4α-cMyc interaction in liver regeneration after drug-and diet-induced liver injury. We found that after acetaminophen-induced acute induced liver injury, HNF4α contributes to regeneration by downregulating the expression of cMyc and supports cytoprotection by inducing Nrf2 activity (11). During choline deficient and ethionine supplemented (CDE) diet-induced chronic liver injury, HNF4α protects against injury, which is exacerbated by cMyc (12). Both the acetaminophen overdose and CDE diet feeding models are different from PHX in the context of liver injury, inflammation, and regenerative cell type. In this study, we investigated the role of HNF4α-cMyc interaction in the regulation of liver regeneration after PHX, a model with significantly less inflammation.

## Materials and methods

### Animal care, surgeries, and tissue harvesting

All studies were approved and performed in accordance with the Institutional Animal Care and Use Committee at the University of Kansas Medical Center. The KUMC IACUC abides by the ARRIVE statement. All animals were housed in facilities accredited by the Association for Assessment and Accreditation of Laboratory Animal Care at the University of Kansas Medical Center under a standard 12 h light/dark cycle with free access to chow and water. Two-month-old male HNF4α-floxed, cMyc-floxed and HNF4α-cMyc double floxed mice were injected with AAV8-TBG-CRE from Vector Biolabs at 2.5E^11^ virus particles dissolved in 300 μL of saline intraperitoneally to generate hepatocyte-specific HNF4α-Knockout (KO), cMyc-KO, and double-KO mice, respectively, and AAV8-TBG-GFP to generate control (WT) mice as previously stated (7). PHX surgeries were performed on these mice one week after AAV8-TBG-CRE injections as previously described (13). After a simple surgical procedure, three out of the five liver lobes (which make up 2/3 of the liver mass) were removed without causing any damage to the remaining two lobes. The liver was able to regrow in size to reach its equivalent mass. Mice were euthanized at various time points between 0 and 14 days after PHX. Liver tissue and blood samples were collected and processed for further analysis. Liver injury was measured using serum ALT activity (Pointe Scientific ALT Assay by Fisher Scientific) by following manufacturer’s protocol.

### Protein isolation and western blotting

Western blot analysis was performed using pooled samples of isolated proteins, as described previously (14). HNF4α (Perseus Proteomics, PP-H1415-00) and cMyc (Cell Signaling Technology, 56055), Cyclin D1 (Cell Signaling Technology, 2978), and GAPDH (Cell Signaling Technology, 2118) antibodies were used. Western blots were imaged using Li-Cor Odyssey FC.

### Real-time polymerase chain reaction

RNA isolation and cDNA conversion were performed as previously stated (14). qPCR reactions were analyzed on the BioRad CFX384 using 100 ng of cDNA per reaction. The 18 s gene expression in the same samples was used to normalize the ct values, as described previously (15). The Ct values for 48 h, 7 days, and 14 days were normalized to the WT mice control at 0 h. The exon spanning primers used for real-time PCR are listed in **Table 1**.

**Table 1:** Primers used in this study.

Gene	Forward Primer (5’-3’)	Reverse Primer (5’-3’)
*Hnf4α*	CAGTGTCGTTACTGCAGGCTT	GCTGTCCTCGATGCTTGACC
*Ccnd1*	GAATTCTATGACCCCTTGACCCC	TGGTGTTGGGTAAGAGGTTG
*F12*	GCCATTTTCCCTTTCAGTACC	TCTTTCACTTTCTTGGGCTCC
*Krt19*	GTTCTCAGACCTGCGTCC	TGACAAAATGCGTACTGAAC
*Epcam*	GCGGCTCAGAGAGACTGTG	CCAAGCATTTAGACGCCAGTTTT
*Col1a1*	ATGTTCAGCTTTGTGGACCTC	CAGAAAGCACAGCACTCGC
*Col1a2*	GGTGAGCCTGGTCAAACGG	ACTGTGTCCTTTCACGCCTTT
*Col3a1*	CCTGGCTCAAATGGCTCAC	CAGGACTGCCGTTATTCCCG
*Clec4f*	TTGGAGACCTGAGTGGAATAAAG	TAGTCCCTAAGCCTCTGGATAG
*Ccr2*	ATGCAAGTTCAGCTGCCTGC	ATGCCGTGGATGAACTGAGG
*Vsig4*	CCCTGGCTTCCTTTCTTCTTA	GCTGTCAGGCATGATAAA
*Adgre1*	CTGCACCTGTAAACGAGGCTT	TTGAAAGTTGGTTTGTCCATTGC
*18s*	ACGGAAGGGCACCACCAGGA	TTTAGTATTGGACGCTGCCC

### Staining procedures

Formalin-fixed and paraffin-embedded liver tissue sections (5 μM thick) were used for H&E, immunohistochemical staining of HNF4α (Perseus Proteomics PP-H1415-00, 1:500), Ki67 (Cell signaling technology 12202, 1:400), and Cyclin D1 (Cell Signaling Technology) and immunofluorescence staining of Epcam (Abcam 71916, 1:500), CK19 (Abcam 52625, 1:600), CYP2F2(Santa Cruz, sc-374540, 1:200) and HNF4α (Perseus Proteomics, PP-H1415-00,1:500) as previously described (14, 16).

### Statistical analysis

Data presented in the form of bar graphs show the mean ± standard error of the mean. GraphPad Prism 9 was used to graph and calculate statistics. Two-way ANOVA, one-way ANOVA, and Student’s *t-test* were applied for all analyses, with *p*<0.05 considered significant. For all the experiments, three to five mice were used per group.

## Results

### Delayed liver regrowth in DKO mice following PHX

**Figure 1:**
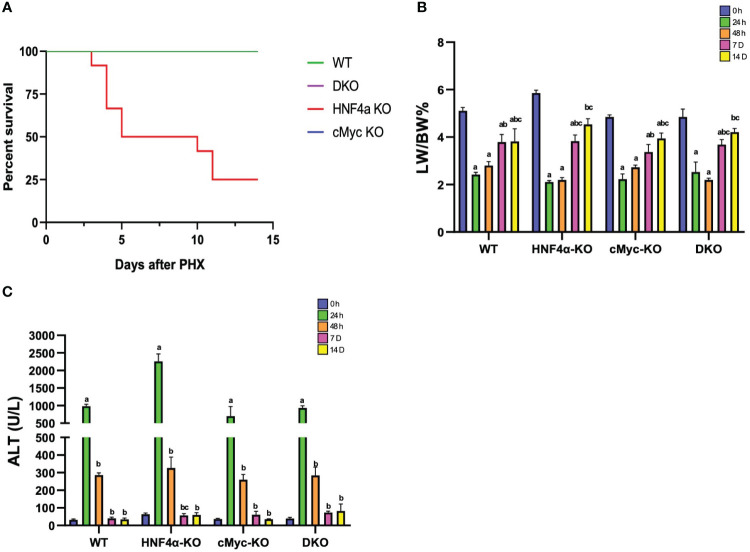
Delayed liver regrowth in DKO mice following PHX. **(A)** Percent survival **(B)** percent LW/BW ratio and **(C)** serum ALT levels in WT, HNF4α-KO, cMyc-KO and DKO mice after 0h, 24h, 48h, 7D, and 14D after PHX. Bars represent means ± SEM. *n* = 3 to 15. Significant change in comparison with 0h=a, 24h=b, and 48h=c.

One of the major findings of our study was that, in contrast to our previous reports, some of the HNF4α-KO mice survived beyond day 10 after PHX (**Figure 1A**). We studied liver regeneration after partial hepatectomy at different time points in WT, HNF4α-KO, cMyc-KO and DKO mice. The liver weight-to-body weight ratio indicated that in WT and cMyc-KO mice, liver regrowth started as early as 48 h following PHX. However, in HNF4α-KO and DKO mice, liver regrowth was delayed, and a significant increase was observed at 7 days post-PHX. Interestingly, all 4 genotypes, WT, HNF4α-KO, cMyc-KO and DKO mice regained liver mass by 14 days post-PHX (**Figure 1B**). All four genotypes (WT, HNF4α-KO, cMyc-KO, and DKO mice) showed an increase in serum ALT levels at 24 h which was significantly lowered by 48 h after PHX (**Figure 1C**). To further investigate the immediately increased injury response in these mice, we performed qPCR analysis of fibrosis markers *Col1a1*, *Col1a2* and *Col3a1* and inflammation markers *Adgre1*, *Clec4f*, *Ccr2* and *Vsig4*. Fibrosis markers were significantly upregulated in HNF4α-KO 7D following PHX. DKO mice showed upregulation in Col1a1 and Col3a1 at 7D time point after PHX. mRNA expression of inflammation markers was significantly upregulated in DKO mice 7D after PHX. Ccr2 expression was increased in HNF4α-KO mice whereas Clec4f and Vsig 4 were upregulated in cMyc-KO mice at 7D time point following PHX (**Supplementary Figures 1A–G**).

### DKO mice show decreased hepatocyte proliferation and disruption in cell proliferation kinetics

**Figure 2:**
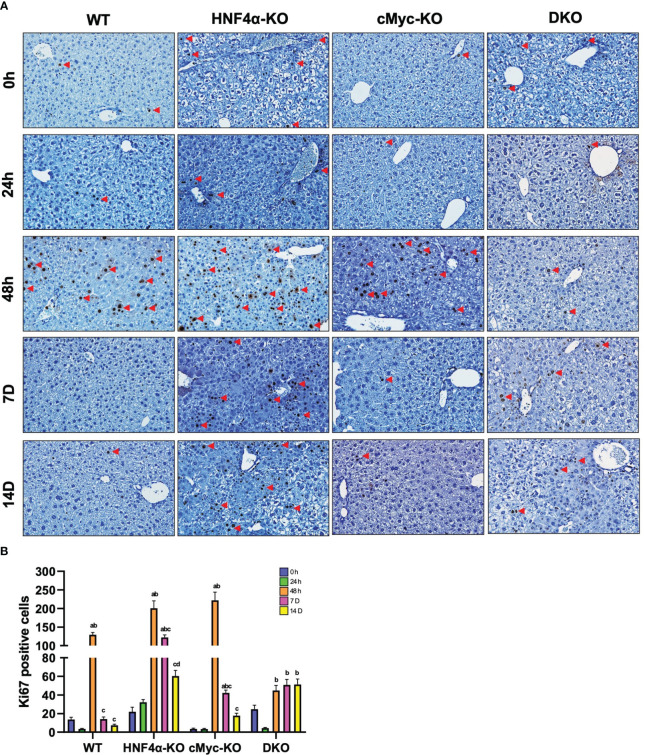
DKO mice show decreased hepatocyte proliferation. **(A)** Representative photomicrographs of Ki67 immunohistochemistry **(B)** Quantification of Ki67 positive cells in WT, HNF4α-KO, cMyc-KO and DKO mice after 0h, 48h, 7D, and 14D after PHX. Bars represent means ± SEM. *n* = 3 to 5. Original magnification 400x. Significant change in comparison with 0h=a, 48h=b, and 7D=c.

**Figure 3:**
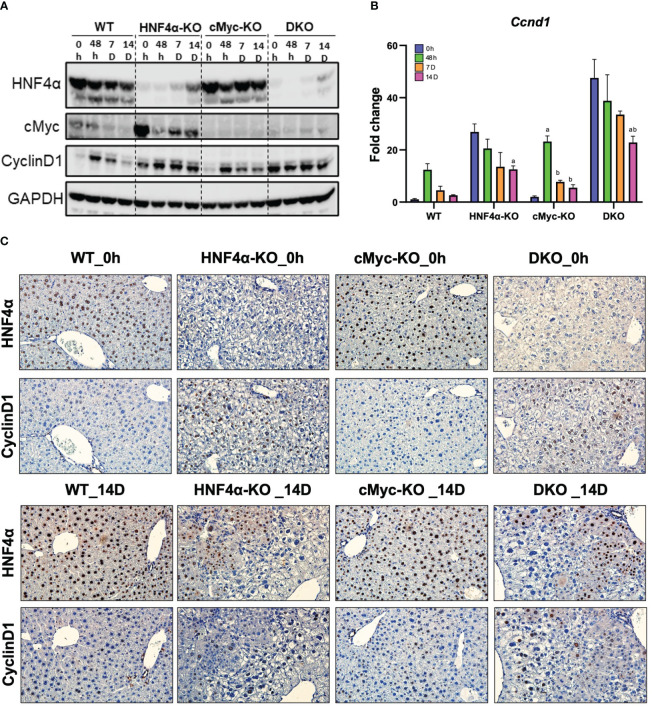
DKO mice show disruption in cell proliferation kinetics. **(A)** Western blot analysis of HNF4α, cMyc and cyclin D1, and **(B)** qPCR analysis of *Ccnd1* in WT, HNF4α-KO, cMyc-KO and DKO mice after 0h, 48h, 7D, and 14D after PHX. **(C)** Representative photomicrographs immunohistochemistry analysis of HNF4α and CyclinD1 in HNF4α-KO and DKO mice 14 days after PHX. Bars represent means ± SEM. *n* = 3 to 5. Original magnification 400x. Significant change in comparison with 0h=a, 48h=b, and 7D=c.

Ki67 immunohistochemical analysis was performed to determine changes in hepatocyte proliferation after PHX. In WT, HNF4α-KO, and cMyc-KO mice, peak cell proliferation was observed at 48 h post-PHX. The number of Ki67 positive cells declined significantly in WT and cMyc-KO mice by day 7 post-PHX. In the HNF4α-KO mice, a decrease in proliferation was observed as compared to 48 h, but proliferation remained significantly higher 7 and 14 days after PHX as compared to all other groups. Interestingly, the DKO mice had significantly lower but sustained cell proliferation as compared to all other genotypes from 48 h to 14 days after PHX (**Figures 2A, B**). Next, we determined changes in cyclin D1, the major cell cycle regulator in liver regeneration. Western blot analysis of cyclin D1 protein indicated a significant increase in HNF4α-KO and DKO mice at all time points after PHX. In the WT and cMyc-KO mice, cyclin D1 protein expression was high at 48 hours after PHX and decreased by 14 days after PHX (**Figure 3A**). qPCR analysis showed higher expression of *Ccnd1* in HNF4α-KO and DKO mice throughout all the time points and peak *Ccnd1* expression at 48 hours after PHX in WT and cMyc-KO mice (**Figure 3B**). Immunohistochemistry analysis of Cyclin D1 confirmed increased expression in HNF4α-KO and DKO mice before and after PHX, consistent with Western blot data (**Figure 3C**).

### Hepatic progenitor cells support regeneration in HNF4α-KO and DKO mice

**Figure 4:**
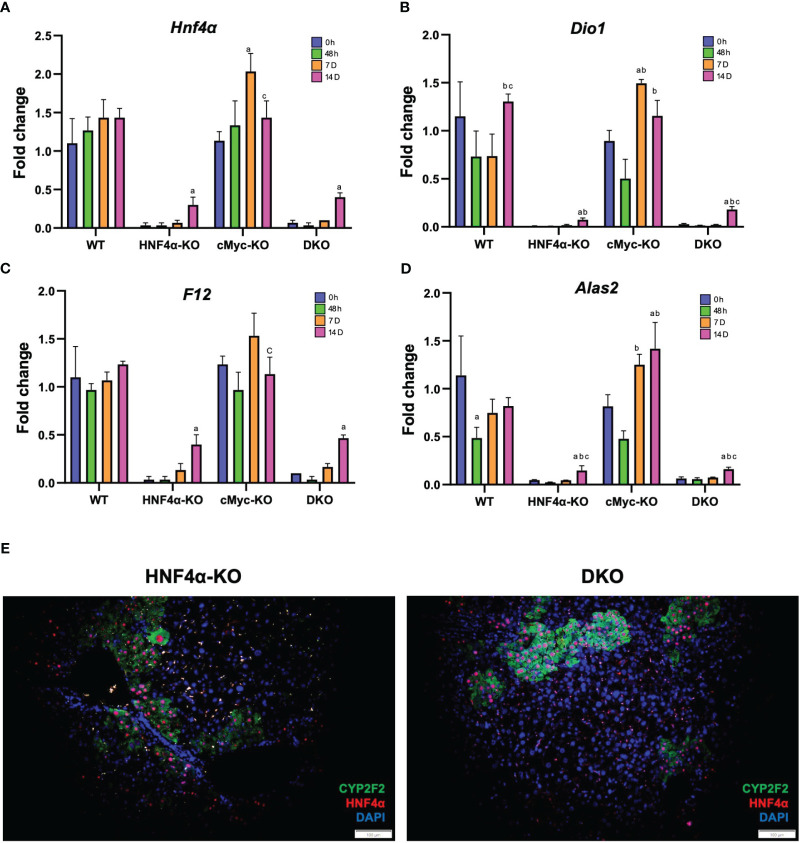
HNF4α re-expression in HNF4α-KO and DKO mice after PHX. qPCR analysis of **(A)** *Hnf4α*, **(B)** *F12*, **(C)** *Dio1*, **(D)** *Alas2* in WT, HNF4α-KO, cMyc-KO and DKO mice after 0h, 48h, 7D, and 14D after PHX. **(E)** Representative photomicrographs of immunofluorescence staining of HNF4α and CYP2F2 in HNF4α-KO and DKO mice 14 days after PHX. Original magnification 400X. Bars represent means ± SEM. *n* = 3 to 5. Significant change in comparison with 0h=a, 48h=b, and 7D=c.

We investigated the mechanism of this increased survival in HNF4α-KO mice. Western blot analysis showed moderate re-expression of HNF4α protein in HNF4α-KO and DKO mice 14 days after PHX (**Figure 3A**). Immunohistochemical staining HNF4α corroborated the Western blot data and showed the appearance of HNF4α positive cells in the HNF4α-KO and DKO mice at 14 days after PHX (**Figure 3C**). These cells were primarily located around portal areas. We performed qPCR analysis of *Hnf4α* and its target genes *F12, Dio1, Alas2.* We observed a significant increase in the expression of these genes in HNF4α-KO and DKO mice at 14 days after PHX compared to WT mice (**Figures 4A–D**). To further confirm if the new HNF4α-positive cells appearing in the HNF4α-KO and DKO mice came from the periportal region, we performed co-staining for HNF4α and the periportal marker CYP2F2. Co-localization of HNF4α and CYP2F2 confirmed that the new HNF4α-positive cells originated from the periportal region (**Figure 4E**).

**Figure 5:**
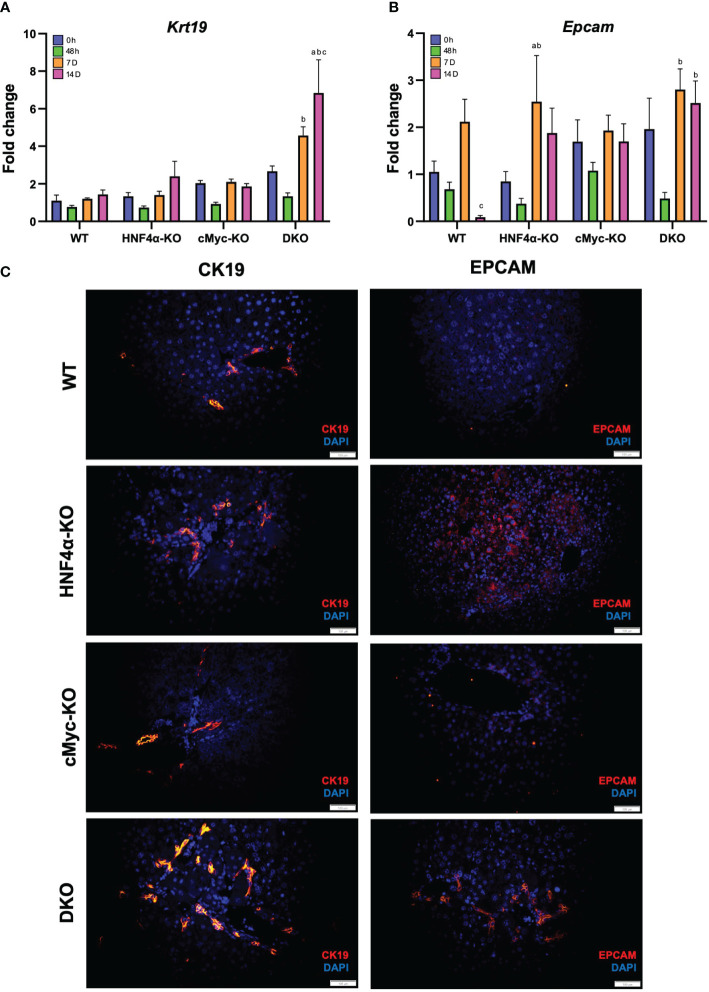
Hepatic progenitor cells support regeneration in HNF4α-KO and DKO mice. qPCR analysis of **(A)** Krt19, and **(B)** Epcam in WT, HNF4α-KO, cMyc-KO and DKO mice after 0h, 48h, 7D, and 14D after PHX. **(C)** Representative photomicrographs of CK19 and EPCAM immunofluorescence staining in WT, HNF4α-KO, cMyc-KO and DKO mice 14 days after PHX. Original magnification 400X. Bars represent means ± SEM. *n* = 3 to 5. Significant change in comparison with 0h=a, 48h=b, and 7D=c.

To analyze whether the newly formed cells are originating from biliary trans-differentiation or the hepatic progenitor compartment, we performed qPCR analysis of hepatic progenitor cell (HPC) markers, which demonstrated a significant increase in mRNA expression of *Krt19* and *Epcam* in DKO mice at 14 days after PHX (**Figures 5A, B**). Immunofluorescence staining of CK19 and EPCAM corroborated with the mRNA data and showed significantly increased staining in HNF4α-KO and DKO mice at 14 days after PHX (**Figure 5B**).

## Discussion and conclusions

HNF4α is a highly conserved nuclear receptor that is considered a master regulator of hepatic differentiation, as HNF4α regulates various metabolic processes (1). The role of HNF4α in proliferation and differentiation is very well established. Deletion of HNF4α has been shown to result in a de-differentiated phenotype and increased proliferation (7). The proliferation phase in liver regeneration is critical, as it allows hepatocytes to enter the G1 phase and helps compensate for the lost tissue (17). One of the major genes upregulated after HNF4α deletion is cMyc, indicating an inverse relationship between HNF4α and cMyc (6). Previous studies have demonstrated that HNF4α and cMyc are functional competitors that regulate p21 expression (18). Studies in our laboratory have been aimed at studying the role of HNF4α-cMyc interaction in liver regeneration using different liver injury models. In the acetaminophen-induced acute liver injury model, we found that the deletion of cMyc in HNF4α-KO mice was protective against the injury (11). In the CDE diet model, which mimics chronic liver injury, and thus the different regeneration process than that after acute liver injury, we found that deletion of HNF4α increased CDE diet-induced injury by contributing to inflammation and fibrotic changes, and deletion of cMyc protects against such injury (12). The PHX model of liver regeneration is different from these two models in the context of injury and regeneration. The PHX model is surgical removal of 70% of the liver tissue, which does not involve massive injury or inflammation response observed in either the acetaminophen overdose or CDE diet model. Our laboratory has previously studied the role of HNF4α in liver regeneration after PHX using hepatocyte-specific HNF4α-KO mice (7). We found that HNF4α regulates cell cycle entry in hepatocytes during the regeneration process, and HNF4α is important for the termination of regeneration. The goal of this study was to investigate the role of HNF4α-cMyc interaction in liver regeneration after PHX.

In our previous PHX study with HNF4α-KO mice from our group, we observed 100% mortality within 10 days after PHX. Our data demonstrated that hepatocytes lacking HNF4α were unable to compensate for HNF4α loss and failed redifferentiation. In our current study, we managed to get three HNF4α-KO mice who survived until day 14 after PHX. HNF4α-KO mice showed significantly higher liver injury immediately after PHX compared to WT control mice. However, there was no increase in fibrosis or inflammation markers. Deletion of HNF4α results in an increase in hepatocyte proliferation and thus an increased liver weight-to-body weight ratio. Consistent with previous studies, we observed a higher LW/BW% ratio in HNF4α-KO mice at the basal level. Interestingly, all genotypes showed similar recovery in LW/BW ratio following PHX. Hepatic deletion of cMyc has been shown not to impair the liver regeneration process after PHX (19). Our data corroborate these data, as hepatocyte-specific cMyc-KO mice showed regeneration patterns similar to WT mice and started gaining liver weight as early as 48 hours after PHX. HNF4α-KO and DKO showed a delay in regeneration response with a significant increase in liver weight at days after PHX. These data indicate that loss of HNF4α or HNF4α and cMyc both together impairs liver regeneration after surgical resection, which is different from what we have reported in either the acetaminophen overdose model or the CDE diet model.

Our data demonstrated that in WT, cMyc-KO, and DKO mice, hepatocyte proliferation started 48 h after PHX. Interestingly, in HNF4α-KO mice proliferation response was high at 24 hours after PHX and a peak proliferation at 48 hours after PHX. Moreover, we observed an overall decrease in Ki67-positive cells and cyclin D1 protein expression in DKO mice after PHX. The peak proliferation at 48 hours after PHX in DKO mice was significantly lower than WT, HNF4α-KO and cMyc-KO mice at the same time point. These data indicate that loss of cMyc along with HNF4α results in a change in the kinetics of cell proliferation rather than the extent of cell proliferation. Because of this, the DKO mice were able to gain similar liver weight by 14 days after PHX when compared to other genotypes.

Interestingly, we observed re-expression of HNF4α at mRNA and protein levels in the HNF4α-KO mice who survived. The newly formed islands of cells expressing HNF4α were exclusively located around the portal triad. We observed a similar re-expression of HNF4α in DKO mice 14 days after PHX. These data suggest that re-expressed HNF4α is important for the survival, recovery of liver weight, and termination of liver regeneration in HNF4α-KO and DKO mice after PHX. HNF4α negatively regulates Cyclin D1 expression_. We observed Cyclin D1 expression exclusively around the central vein in HNF4α-KO and DKO mice 14 days after PHX. The newly formed cells near portal triad expressing HNF4α did not express Cyclin D1. Co-staining of HNF4α with periportal marker CYP2F2 further confirmed the periportal origin of newly formed cells in these mice. Further, the expression of the HPC markers was significantly higher in DKO mice 14 days after PHX. This suggests that liver regeneration in DKO mice not only includes hepatocyte proliferation but also HPC proliferation. Hepatocytes predominate the regeneration response after acute liver injury and PHX (20). cMyc compensates for hepatocyte proliferation in the absence of HNF4α during liver regeneration after PHX. However, cMyc deletion in HNF4α-KO mice promotes HPC-driven proliferation response to regain liver growth. These data further highlight the role of HPCs as a secondary mechanism in liver regeneration. The exact signals that drove the HPC activation in the surviving HNF4α and DKO mice are not clear at this time.

In summary, HNF4α is critical for the survival and termination of liver regeneration after PHX. In the absence of HNF4α, cMyc contributes to hepatocyte-driven proliferation to compensate for the lost tissue mass. In the absence of both HNF4α and cMyc, HPC-driven proliferation occurs.

## Data availability statement

The raw data supporting the conclusions of this article will be made available by the authors, without undue reservation.

## Ethics statement

The animal study was approved by IACUC of the University of Kansas Medical Center. The study was conducted in accordance with the local legislation and institutional requirements.

## Author contributions

UA: Conceptualization, Data curation, Funding acquisition, Investigation, Methodology, Project administration, Resources, Supervision, Writing – review & editing. MK: Data curation, Methodology, Supervision, Writing – original draft, Writing – review & editing. DP-C: Investigation, Methodology, Writing – review & editing. KV: Investigation, Writing – review & editing.

## Supplementary material

Supplementary Figure 1 Fibrotic and Inflammatory changes after PHX. qPCR analysis of **(A)** Col1a1, **(B)** Col1a2, **(C)** Col3a1, **(D)** Adgre1, **(E)** Clec4f, **(F)** Ccr2, **(G)** Vsig4 in WT, HNF4α-KO, cMyc-KO and DKO mice after 0h, 48h, 7D, and 14D after PHX. Bars represent means ± SEM. *n* = 3 to 5. Significant change in comparison with 0h=a, 48h=b, and 7D=c.
