# Supplementary figures and images for: Role of HNF4alpha-cMyc interaction in liver regeneration after partial hepatectomy
Source: Front Endocrinol (Lausanne). 2024 Jul 31;15:1404318. doi: 10.3389/fendo.2024.1404318 (PMC11322135; doi:10.3389/fendo.2024.1404318)

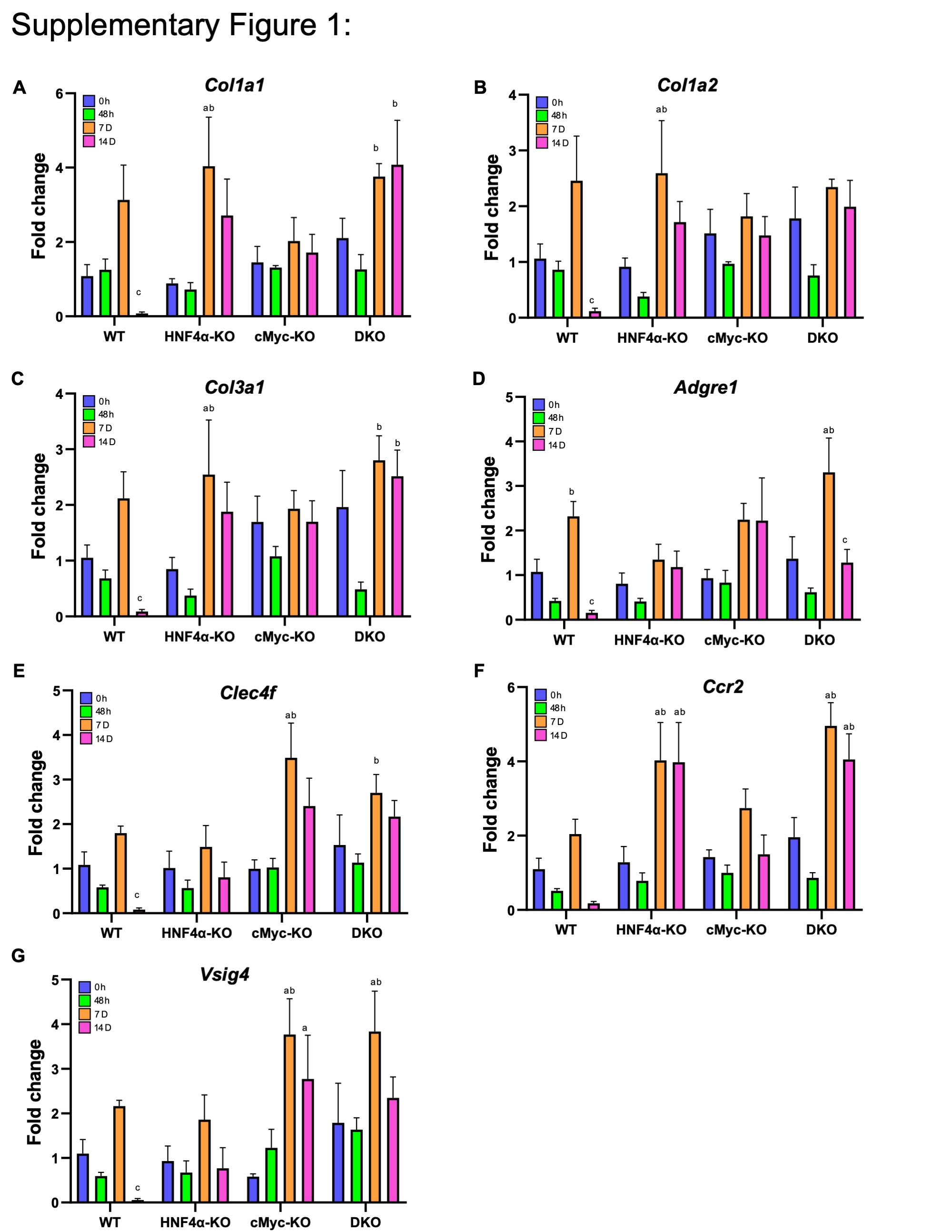

Supplement: Supplementary Figure 1 — Fibrotic and Inflammatory changes after PHX. qPCR analysis of (A) Col1a1, (B) Col1a2, (C) Col3a1, (D) Adgre1, (E) Clec4f, (F) Ccr2, (G) Vsig4 in WT, HNF4α-KO, cMyc-KO and DKO mice after 0h, 48h, 7D, and 14D after PHX. Bars represent means ± SEM. n = 3 to 5. Significant change in comparison with 0h=a, 48h=b, and 7D=c. [file Image_1.jpeg]
